# Supplementary material for: Content-rich biological network constructed by mining PubMed abstracts
Source: BMC Bioinformatics. 2004 Oct 8;5:147. doi: 10.1186/1471-2105-5-147 (PMC528731; doi:10.1186/1471-2105-5-147)
Supplement: Additional File 2 — The original results of the above study (non-essential files are deleted to keep the file size under the limit set by BMC bioinformatics). [file 1471-2105-5-147-S2.bz2 › chilibotAdditionalFile2/dip10/235ID8006584E759/html/IL2RB_IL2.html]

 


 **IL2RB** and **IL2** 
  
Found 3819 abstracts in PubMed, retrieved 10.  
 

 What does Google say? 
 PDF only 
| .edu only 

---

**Interactive relationship** (e.g. stimulation, inhibition, etc)

**Non-interactive relationship** (e.g. studied together, co-existance, homology, etc.)

- Immunosuppression protocol anti IL 2  [ **IL2** ]  receptor  [ **IL2RB** ]  antibodies, steroids, mycophenolate mofetil MMF.  Ref: 12876872 Acta Med Croatica, 2003
- Addition of IL 12 to this coculture prevented apoptosis of the CD4 T cells, which was associated with up modulation of IL 2  [ **IL2** ]  receptor  [ **IL2RB** ]  beta chain expression.  Ref: 12883484 Hepatology, 2003
- The suppressive activity of CD8 CD25 thymocytes was abrogated by a mixture of anti CTLA 4 and anti TGF beta1 antibodies and it was mediated by their ability to inhibit the expression of the IL 2  [ **IL2** ]  receptor  [ **IL2RB** ]  alpha chain on target T cells.  Ref: 12893750 BloodBlood, 2003
- Although the effects of mitogens on the synthesis of interleukin 2  [ **IL2** ]  IL 2  [ **IL2** ]  and IL 2  [ **IL2** ]  receptor  [ **IL2RB** ]  IL 2r have been described, a detailed in situ analysis of the spatio temporal changes of the expression of the IL 2  [ **IL2** ]  gene and the three IL 2r components in lymphoid tissues is still missing.  Ref: 12890448 Cytokine, 2003
- Most investigated Treg cells are CD4 pos cells expressing the alpha chain of the IL 2  [ **IL2** ]  receptor  [ **IL2RB** ]  CD25.  Ref: 12848995 Autoimmun Rev, 2002
- Plasticity of IL 2  [ **IL2** ]  and IL 2  [ **IL2** ]  receptor  [ **IL2RB** ]  chains in rat lymphoid tissues in situ after stimulation with staphylococcal enterotoxin A.  Ref: 12890448 Cytokine, 2003
- The pathological changes of the graft, IL 2  [ **IL2** ]  receptor  [ **IL2RB** ]  expression in lamina propria lymphocytes, serum IL 2  [ **IL2** ]  concentrations, results of spleen lymphocytes transformation test and the IL 2  [ **IL2** ]  secretion capacity were determined and compared.  Ref: 12875685 Chin Med J (Engl), 2003
- The concentration of soluble IL 2  [ **IL2** ]  receptor  [ **IL2RB** ]  R protein was measured in BAL fluid using ELISA as well, as an indicator of lymphocyte activity.  Ref: 12869037 Clin Exp Immunol, 2003
- We detected an upregulation or de novo synthesis of interleukin 1alpha IL 1alpha, IL 1beta, IL 8, macrophage inflammatory protein 1alpha MIP 1alpha, MIP 1beta, GRO alpha, transforming growth factor beta, MCP 1, IL 2  [ **IL2** ]  receptor  [ **IL2RB** ]  gamma IL 2Rgamma, IL 10Ralpha, and IL 6R.  Ref: 12874345 Infect Immun, 2003
- Presentation by these migrants sustained expression of the IL 2  [ **IL2** ]  receptor  [ **IL2RB** ]  and promoted delayed type hypersensitivity.  Ref: 12871638 Immunity, 2003
- We found a defective T cell response in malnourished children, as indicated by diminished levels of circulating total CD3, helper CD4, IL 2  [ **IL2** ]  receptor  [ **IL2RB** ]  bearing CD4 CD25 and memory helper T cell responses CD4 CD45RO in keeping with the decreased specific IgE levels against Ascaris lumbricoides.  Ref: 12848201 J Trop Pediatr, 2003
